# Direct Measurement of the Ciliary Sulcus Diameter Using Optical Coherence Tomography—Inter-Rater Variability

**DOI:** 10.3390/s24216950

**Published:** 2024-10-29

**Authors:** Timo Eppig, Manuel Seer, Antonio Martinez-Abad, Virgilio Galvis, Saskia Schütz, Alejandro Tello, Michiel C. Rombach, Jorge L. Alió

**Affiliations:** 1 Institute of Experimental Ophthalmology, Saarland University, 66421 Homburg, Germany; 2 AMIPLANT GmbH, 91220 Schnaittach, Germany; 3 Vissum Grupo Miranza, 03016 Alicante, Spain; 4 Centro Oftalmologico Virgilio Galvis, Floridablanca 681004, Colombia; 5 Department of Ophthalmology, Faculty of Health, Universidad Autónoma de Bucamaranga (UNAB), Bucamaranga 680002, Colombia; 6 Department of Surgery, Faculty of Health, Universidad Industrial de Santander (UIS), Bucamaranga 680002, Colombia; 7 AKKOLENS International b.v., 4836 BA Breda, The Netherlands

**Keywords:** ocular dimensions, optical coherence tomography, intraocular lens

## Abstract

The determination of sulcus-to-sulcus measurements has been challenging due to the limitations of current approaches. Ultrasound methods are highly operator-dependent and require extensive training, while traditional optical devices cannot visualize structures posterior to the iris. However, modern optical anterior segment coherence tomography (AS-OCT) devices are changing this paradigm by identifying some anatomical landmarks posterior to the iris. This study evaluates the reproducibility of optical sulcus measurements in the context of sizing a novel accommodative intraocular lens (IOL). Preoperative OCT scans of patients scheduled for cataract surgery were analyzed regarding the dimensions of the ciliary sulcus using a custom scan method with a clinically available anterior segment optical coherence tomographer. Measurements were compared between two different readers, and various derived parameters were compared. The measurements by both readers were highly correlated (R^2^ > 0.96), and their agreement was excellent (mean difference 0.02 mm with 95% limits of agreement from −0.11 to 0.15 mm). In contrast, the sulcus diameter measurement did not agree well with automatically calculated values, such as the anterior chamber width or white-to-white. This leads to the conclusion that modern swept-source AS-OCT measurements of the ciliary sulcus dimensions are feasible, reproducible, and may be a clinically useful tool.

## 1. Introduction

**Figure 1:**
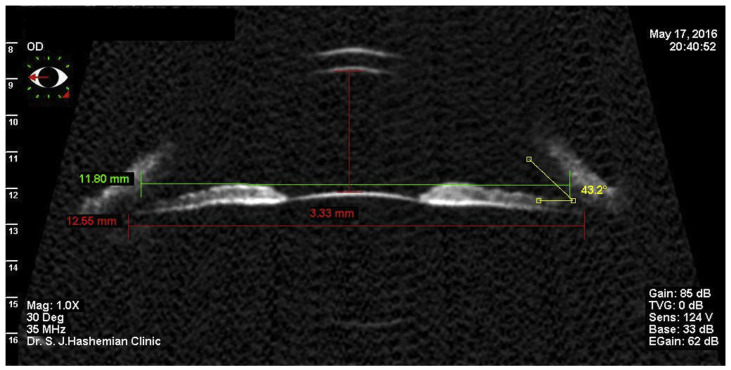
An ultrasound biomicroscopy (UBM) image showing a ciliary sulcus measurement. (Adopted from Hashemian et al. [17] under Creative Commons License).

Posterior chamber phakic intraocular lenses (pIOLs) have become extremely popular in refractive surgery for the correction of moderate to high hyperopia or myopia with and without astigmatism [1]. These are thin lenses made from flexible acrylic polymer. The most popular type, the Visian ICLs (Staar Surgical Inc., Monrovia, CA, USA), are made from proprietary Collamer material, which is flexible. The haptics of these pIOLs are implanted into the ciliary sulcus, preserving the accommodative function of the crystalline lens. The sizing of the posterior chamber pIOLs and their relation to the sulcus-to-sulcus distance is critical, as it determines the postoperative vault, which must be within a specific range. If the vault is too low, alterations in the crystalline lens’ metabolism may occur, potentially leading to cataract formation. If it is too high, the anterior chamber angle could close, causing secondary glaucoma [1,2,3]. The correct sizing of phakic lenses is crucial prior to implantation in order to reduce the risk of increased lens vaulting and anterior angle closure or cataract formation due to contact with the crystalline lens. Since the size of the sulcus cannot be measured directly, the state-of-the-art method for sizing pIOLs is using other ocular parameters in order to estimate the sulcus dimensions. Manufacturers of such pIOLs like Staar Surgical use proprietary nomograms for estimating the proper pIOL size. There have been numerous methods proposed in the literature for sizing prior to the implantation of posterior chamber pIOLs [4,5,6,7,8,9,10,11]. These include an estimation of sulcus-to-sulcus (STS) diameter using the limbal diameter, often referred to as ‘white-to-white’ (WTW) or the aqueous diameter, often referred to as anterior chamber width (ACW). However, Pop et al. showed that WTW alone is not sufficient for the estimation of STS and proposed a formula employing corneal power [12]. Other methods use ultrasound (US) to directly measure the sulcus diameter, operating at frequencies around 20–50 MHz (ultrasound biomicroscopy—UBM) or using very-high-frequency (VHF) digital ultrasound arc-scanners with a broadband 50 MHz ultrasound transducer (bandwidth 10 to 60 MHz) that acquires B-scans of the anterior segment. These approaches are considered to be the ’gold standard’ [13,14,15,16,17,18,19]. Ultrasound is unaffected by optical absorption and is able to penetrate behind light-blocking structures such as the iris. However, being contact methods, they are rather uncomfortable for the patient, and the achievable precision highly depends on the examiner’s experience and the frequency of the ultrasound probe. Ocular imaging and sulcus measurement with UBM are typically performed with 35 MHz or 50 MHz probes [4,5,6,7,8,9,20,21,22], which differ in achievable resolution, while VHF devices are less common in clinical use. Ultrasound imaging is a measurement method using immersion where a funnel is placed into the eye, which can be filled with water. The probe is then immersed in water, limiting the operators ability to detect the measurement location. Sulcus size and ICL vault are then measured by means of digital rulers, as shown in Figure 1. Depending on the operating frequency and integrity of the probe, images are typically more or less noisy, and the lacking coordinate image registration does not allow us to define whether the scan was carried out along the desired meridian/secant.

Almost two decades ago, anterior segment optical coherence tomography (AS-OCT) has been introduced in ophthalmology, offering new diagnostic capabilities for the anterior segment of the eye. The latest developments in swept-source OCT provide increased penetration depth, allowing imaging to the posterior pole of the crystalline lens [23,24]. However, unlike in UBM, the ciliary sulcus is invisible in most images generated by optical tomography due to the limited penetration depth of the OCT beam and the absorptive properties of the posterior iris epithelium, which contains a layer of heavily pigmented columnar cells. Malyugin et al. modified the method previously proposed by Piñero et al. using the posterior pigment epithelium of the iris as a marker for indirect determining sulcus size [5,25]. They developed an algorithm considering the distance between the outer ends of the posterior iris pigment layer [5]. Piñero et al. also demonstrated the correlation between AS-OCT and ultrasound measurements while pointing out that they cannot be used interchangeably [25]. However, this method was developed and tested with a time-domain OCT (Visante OCT, Carl Zeiss Meditec AG, Jena, Germany) operating at a wavelength of 1310 nm, which is optimal for deep penetration into the anterior chamber. Direct transfer to different OCT types and devices is difficult, as the required settings have to be re-investigated. In addition, Piñero et al. did not measure the ciliary sulcus but the anterior chamber width [25], which is a different parameter. We will demonstrate the correlation between anterior chamber width and ciliary sulcus size in this paper. In addition, the Visante OCT used by Malyugin et al. is no longer available on the market, which raises the question of how this method could be transferred to contemporary diagnostic instrumentation. Its successor, the Cirrus 6000 OCT (Carl Zeiss Meditec AG, Jena, Germany), operates at shorter wavelengths, which are better for retinal imaging. New swept-source OCT has been shown to provide superior penetration depth and image quality offering new diagnostic possibilities. Strikingly, no additional studies on the use of AS-OCT to determine the sulcus-to-sulcus distance have been published in the recent nine years [10,26,27,28].

Zhang et al. showed good agreement between Visante OCT and the CASIA SS-1000 OCT (Tomey Corporation, Nagoya, Japan) for measurements within the anterior chamber [27]. Moshirfar et al. stated that the use of OCT-based sizing has improved the overall safety of pIOL surgery in the United States [29].

**Figure 2:**
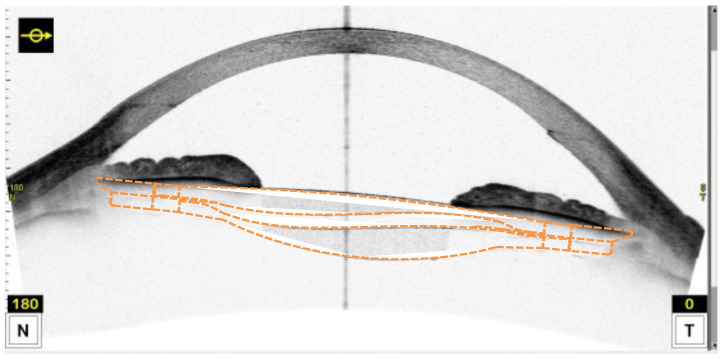
Illustration of the situation after implantation of a Lumina IOL (dashed orange line) into the ciliary sulcus. Centripetal compression of the proprietary haptics induces a lateral shift of the two optic pieces of the lens perpendicular to the optical axis.

The novel Lumina potentially accommodative IOL (Akkolens International B.V., Breda, The Netherlands) is a double-optic system, based on the Alvarez principle which implements two lenses being displaced in a perpendicular movement to the optical axis [30,31]. The mechanism is driven by direct action from the ciliary body, and then the haptics of this accommodative IOL are placed in the ciliary sulcus [32]. The IOL is therefore manufactured to fit an individual eye’s sulcus diameter, as shown in Figure 2. Consequently, the measurement of the sulcus diameter is a crucial part of preoperative examinations in eyes undergoing Lumina IOL implantation.

The challenge was to optimize available parameter settings in a clinical AS-OCT device to establish a reliable method to visualize anatomical structures of the ciliary sulcus by means of optical coherence tomography to facilitate direct sulcus-to-sulcus measurements, compared to the US measurement, rather than using automated anterior chamber width or white-to-white calculation or nomograms to predict the sulcus size. Taking into account that the iris pigment absorbs and/or scatters most of the light, it was uncertain whether sulcus structures can be visualized with sufficient contrast to allow reliable measurements. Previous methods like the one proposed by Piñero and Malyugin [5,25] were limited as the could only show the iris pigment but not the ciliary pigment lying behind. Moreover, they could not show the ciliary muscle. The latest AS-OCT using 1310 nm wavelength, however, offers higher penetration depth and allows for the imaging of the pigment layer of ciliary structures and the ciliary muscle itself [26,33,34]. The purpose of this study was to develop a method for estimating the dimensions of the ciliary sulcus using the posterior pigment epithelium of the iris and ciliary structure as observed in AS-OCT, based on the method proposed by Malyugin et al. [5]. The novelty in this paper is based on an optimized parameter setting of the OCT to increase image quality in order to improve the information in the data.

## 2. Materials and Methods

### 2.1. Patients

In this retrospective clinical evaluation, we included 53 eyes of 28 patients. All patients were scheduled for cataract surgery and implantation of a sulcus fixated Lumina IOL. All patients received a thorough ophthalmogical examination as well as IOLMaster biometry (IOLMaster 500 or 700, Zeiss Meditec AG, Germany) and AS-OCT (CASIA2, Tomey Corp., Nagoya, Japan). Informed written consent was obtained from all patients, and regulatory clearance was obtained from the local ethical review boards.

### 2.2. Measurement Method

The OCT protocol included a standard corneal topography scan (Corneal Map), a global 3-dimensional scan (AS Global Scan) and a set of custom 2-dimensional scans with a high resolution and 64-fold averaging of the slices. This was performed to reduce the noise in the images in order to visualize ciliary body structures. The examinations were performed by the clinical staff and the raw OCT data were transferred in the proprietary export format for further analysis. Measurements were taken with the AS-OCT CASIA2 (Tomey Corp., Nagoya, Japan). The machine uses swept-source technology with a central wavelength of λ = 1310 nm. The axial/lateral resolution in tissue is specified at 10 µm/30 µm. The machine takes up to 50,000 A-scans per second.

**Figure 3:**
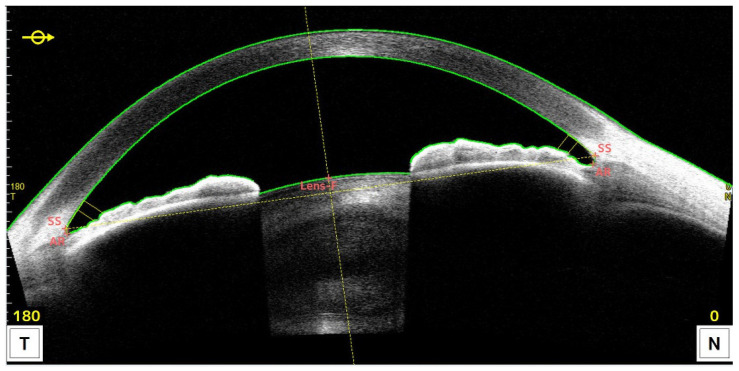
Exemplary anterior segment scan (CASIA2) of the horizontal meridian with standard white on black color space. The surfaces of the cornea and the lens are detected and highlighted by green lines. The ciliary sulcus is hardly visible, as expected, due to the absorption of light by the posterior pigment epithelium of the iris.

**Figure 4:**
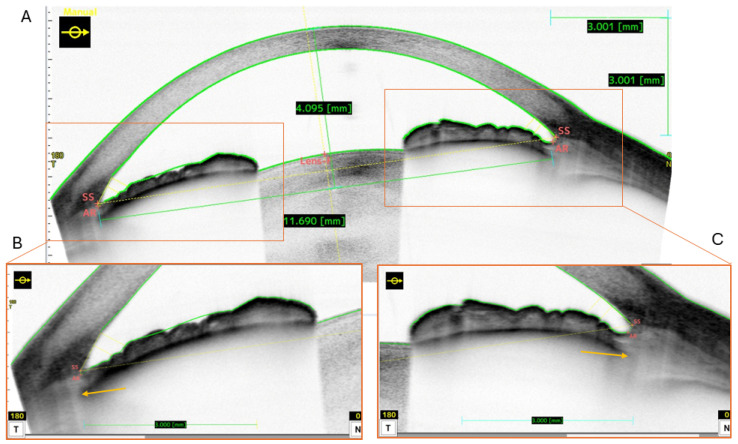
(**A**) Two -dimensional analysis screen of the CASIA2 software (Version 50.7x) showing the overview of the ocular scan and depicting the measurement method for sulcus to sulcus (STS) and sulcus plane depth (SPD) with the two green rulers. The STS measurement was performed manually from the visual interpretation of the ciliary mass. The automated values such as anterior chamber width (ACW) were also recorded, and angle-to-angle (ATA) measurements were additionally logged in the tabulated output format. (**B**,**C**) Magnified regions of the ciliary structure giving an impression of the pigmented structures and the ciliary sulcus (the scale bar is 3.0 mm). The orange arrows show the estimated location of the sulcus.

We defined custom scan methods to determine the size of the ciliary sulcus by measuring the distance from pigment to pigment in the horizontal direction, which is the preferred implantation direction for phakic intraocular lenses and for the Lumina. A set of 3 scans was performed for each eye at 0°, 30° and 150°. A-scan averaging of 64 was used to reduce the noise in the image. Figure 3 shows an exemplary scan of an eye for the 0° direction. The ciliary structure was not visible with the standard settings and the white-on-black color space (Figure 3), but the iris pigment epithelium could be identified. This could be improved by adjusting brightness and contrast in the image (Figure 4 and Table 1), revealing pigmented structures on the ciliary body. The OCT software (Version 50.7x) corrected the image distortion caused by the optical refraction in the image after having detected the corneal surfaces. Position of scleral spur and angle recess points were corrected where the automatic algorithm had failed to identify them correctly.

We then used the integrated software caliper in the 2D analysis mode of the OCT’s software to measure the distance between left and right sulcus (sulcus to sulcus, STS), represented by the outmost dark area (pigment), which was visually identified from the image in Figure 4. We checked the validity of this caliper method by analyzing the geometric data of an IOL post implantation. The depth of the sulcus plane (SPD) was measured as the distance of the line connecting the sulcus to the anterior corneal surface (Figure 3). The STS and SPD values are the required ones to determine the required size of the Lumina implant (STS) and the estimated postoperative IOL position and power (SPD).

Measurements were then saved, and the data were exported to a text file, which contained additional values that were used for further analysis: Firstly, the anterior chamber width (ACW), which is defined as the distance between left and right scleral spur (SS). Secondly, the angle-to-angle (ATA) measurement, which is defined as the distance between left and right angle recess points (AR). The difference between these parameters can be derived from Figure 3. Both are being measured in the anterior chamber. In addition, we measured the white-to-white (WTW) value with the integrated software tool. All measurements were performed by two trained operators (TE & MS). If one of the slices was unavailable in high resolution, the measurement was obtained from the global scan.

From these three values, we calculated the mean value and standard deviation (SD). In addition, we calculated the correlations between the sulcus measurement and anterior chamber width as well as the white-to-white parameter and sulcus depth with anterior chamber depth derived from the IOLMaster (IOLMaster 500 or 700, Zeiss Meditec AG, Jena, Germany). All values were recorded in a Microsoft Excel 365 (Microsoft Corp., Redmond, WA, USA) spreadsheet for statistical analysis.

### 2.3. Validity

In an instance where we were lacking a paired sample of ultrasound measurements or a gold-standard phantom, we used postoperative OCT images from 12 eyes of 9 patients that underwent implantation of the Lumina IOL. We analyzed the diameter of the posterior lens because the anterior lens was less visible (compare Figure 2). The data were correlated with manufacturing records of the lenses, whilst taking manufacturing tolerances (0.05 mm) and OCT image resolution into account.

### 2.4. Statistical Analysis

Statistical analysis was performed with SPSS (IBM Corp., Armonk, VA, USA) and MATLAB R2023b (The MathWorks Inc., Natick, MA, USA). We calculated the mean and standard deviation of the STS, SPD, ACW and ACW. Statistical differences between STS, ACW and WTW were analyzed in a signed-rank sum test. Then, we correlated the CASIA2 parameters to corresponding parameters derived from the IOLMaster. We used MATLAB R2023b for the data analysis and for generating the graphs. We compared STS measurement to the suspected predictors by calculating Pearson’s correlation coefficient and by performing Bland-Altman analysis [35].

## 3. Results and Discussion

The preoperative characteristics of the 53 included eyes are shown in Table 2. The descriptive data for these patients and readings of both readers is shown in Table 3.

### 3.1. Validity

**Figure 5:**
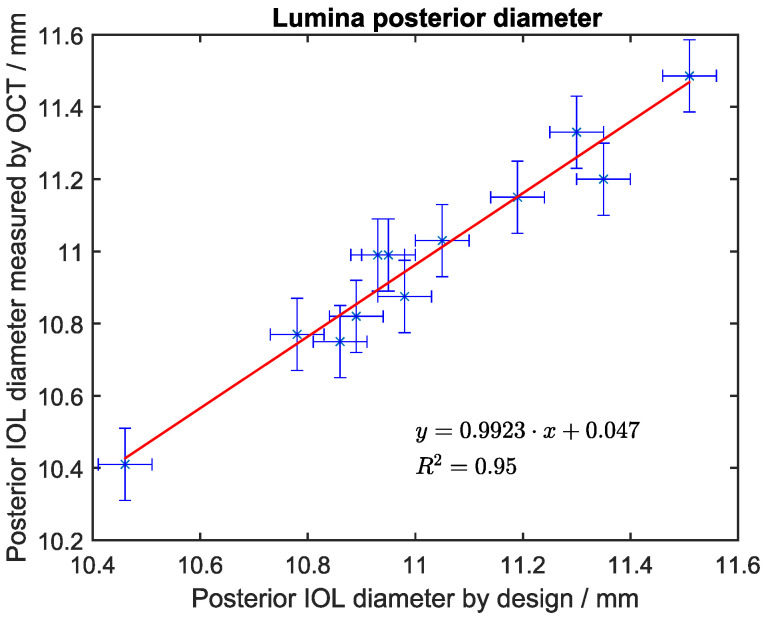
Correlation between for the posterior lens diameter of the Lumina IOL from manufacturing records and the postoperative measurement using OCT. The error bars depict the manufacturing tolerance (horizontal) and the estimated OCT measurement accuracy (vertical).

The correlation between OCT measurement and manufacturing records for the lenses was excellent, with an R^2^ of 0.95 (Figure 5).

### 3.2. Inter-Rater Repeatability

**Figure 6:**
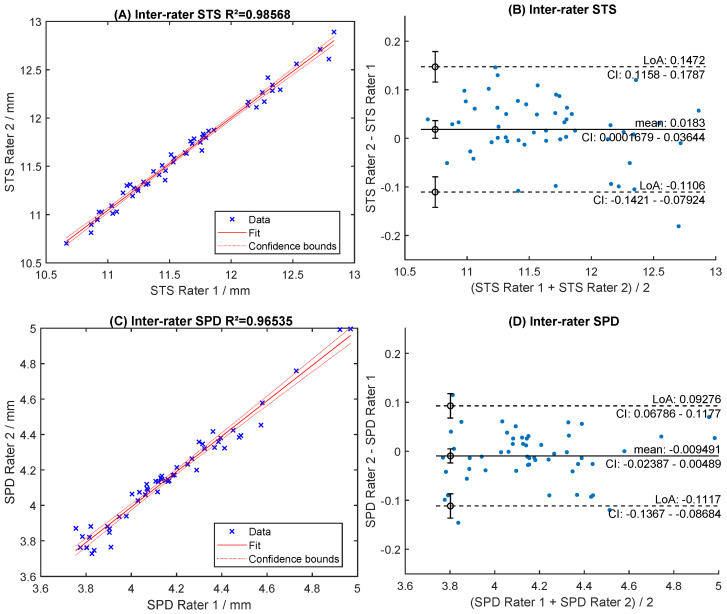
Inter-rater correlation and Bland-Altman levels of agreement for sulcus-to-sulcus (STS) measurement (**A**,**B**) and sulcus plane depth (SPD) (**C**,**D**), respectively.

**Figure 7:**
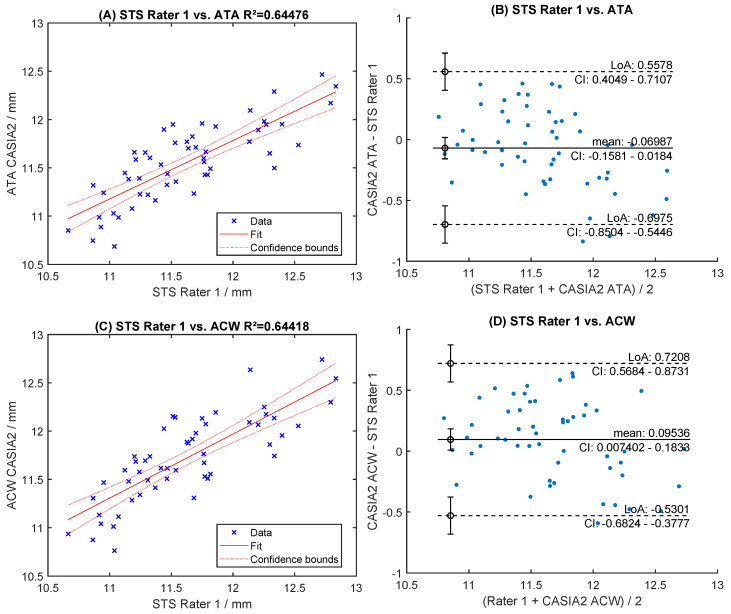
Inter-rater correlation and Bland-Altman levels of agreement for anterior chamber width (ATA) (**A**,**B**) and angle-to-angle (ACW) measurement (**C**,**D**), respectively.

**Figure 8:**
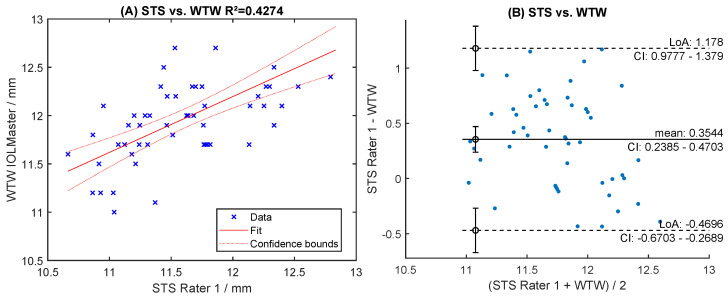
Correlation and Bland-Altman levels of agreement for sulcus-to-sulcus (STS) with white-to-white (WTW) measurements (**A**,**B**).

**Figure 9:**
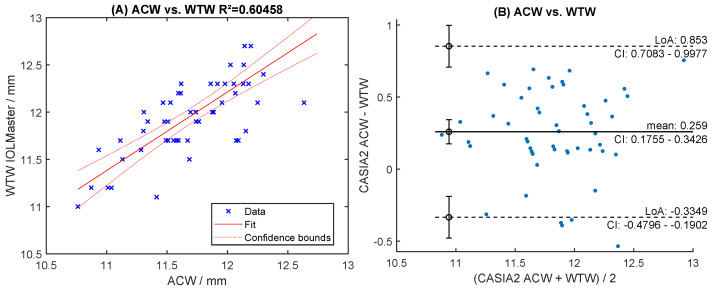
Correlation and Bland-Altman levels of agreement anterior chamber width (ACW) with WTW (**A**,**B**).

**Figure 10:**
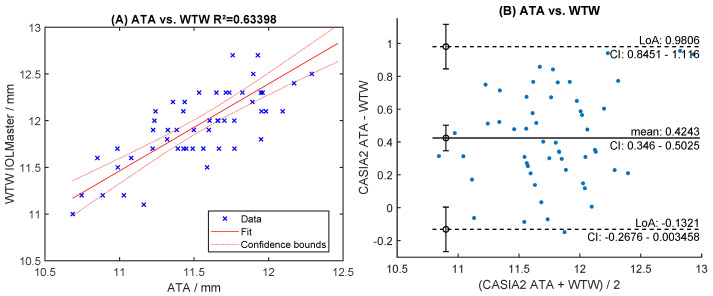
Correlation and Bland-Altman levels of agreement for angle-to-angle (ATA) measurements with WTW (**A**,**B**).

Readings of both raters were highly correlated (R^2^ > 0.96, Figure 6A,C). The mean difference was 0.018 mm for STS and −0.011 mm for SPD (Figure 6B,D). The levels of agreement were within [−0.109; +0.145] mm for STS and [−0.112; +0.091] mm for SPD (Figure 6B,D). Manual readings of STS correlated poorly with automated ACW and ATA measurements (R^2^ < 0.66, Figure 7A,C). The Bland-Altman analysis showed large levels of agreement (Figure 7B,D). Correlation with WTW to any of the sulcus measurements (STS, ACW, ATA) was very poor (R^2^ < 0.63, Figure 8A, Figure 9A and Figure 10A), with large levels of agreement (Figure 8B, Figure 9B and Figure 10B).

### 3.3. Discussion

In the present study, we investigated the reliability of a method using anterior segment OCT images to determine the diameter of the ciliary sulcus. In standard imaging settings, the ciliary sulcus is hardly visible due to the high absorption of visible and near infrared light in the iris pigment. Ciliary sulcus structures could be visualized using an optimized set of parameters. Wagner et al. have shown that AS-OCT is capable of imaging the ciliary muscle when focusing directly on the ciliary body (e.g., in dedicated angle measurement) [33]. However, to the best of our knowledge, this is the first study—apart from Malyugin et al.—to investigate the diameter of the ciliary sulcus with AS-OCT [5]. Malyugin et al. used a method based on image brightness and defined the sulcus diameter as the distance between the extremal ends of the iris pigment layer [5]. This was based on the methods proposed by Piñero et al., who measured the anterior chamber width with OCT and compared it with STS measurements acquired using VHF ultrasound [25]. Anatomical images, however, show that the sulcus may be even wider than the width of the iris pigment layer. This can be confirmed by the images of eyes with reduced pigment in the outer iris segments and eyes with congenital aniridia, which allow better image quality due to the absence of light absorption. In addition, the choice of imaging instrumentation seems to be crucial. Malyugin used the Zeiss Visante OCT operating at 1310 nm, which is no longer available. Many OCT systems operate at shorter wavelengths, which are optimal for retinal imaging but can also be used for corneal imaging using additional objective lenses. Examples of these include the Zeiss Cirrus OCT, the Heidelberg Engineering Spectralis OCT, and the Optovue Revo. There are only a few systems on the market dedicated for anterior segment imaging and operating at 1310 nm, which allows for deeper penetration and, therefore, higher image contrast in the irido-ciliary complex. One of them is the Tomey CASIA2 AS-OCT, which was used in the current study. The purpose of this study was to find and evaluate a strategy to measure the dimensions of the ciliary sulcus with AS-OCT. Therefore, we used the internal options for defining custom scan types in CASIA2 software (Version 50.7x) in order to improve image contrast. From our previous experience with the device, we already knew that the compensation for corneal refraction was valid, leading to realistic dimensions when measuring distances within the eye [24]. Therefore, we used the internal measurement option for measuring the size of the ciliary sulcus as the distance from pigment to pigment and the depth of the sulcus plane from the corneal apex. However, the measurement requires experienced persons to identify the end point of the pigmented structure and to define the location of the sulcus. Therefore, we had two independent raters analyzing the data. The results showed that the readings of the two were statistically different, but the correlation was high and the Bland-Altman analysis revealed a difference within the resolution of the device. Hence, we conclude that the sulcus reading is independent of the reader. Other ocular measures such as ACW, ATA or WTW show only a poor correlation with the STS measurement and the Bland-Altman analysis, revealing that the mean difference is within a millimeter scale (>0.5 mm). Likewise, Piñero et al. demonstrated the poor correlation of WTW to ACW [25], and Hashemian et al. showed that STS measured by UBM is significantly different from WTW measured with the Orbscan [17]. We conclude that these values should not be used for calculating sulcus size as the variability is too large. This may also be the reason for the high amount of sizing errors with phakic IOLs using conventional formulas. Hashemian et al. proposed a correction formula to calculate STS from WTW for phakic IOL size calculation, but the 95% levels of agreement were still in the range of ±0.6 mm [17].

We also found a difference between ACD and SPD, which can easily be explained in the eyes with thick, protruding lenses, where the ACD, measured from the corneal apex to lens apex, was significantly shorter that the distance between the corneal apex and sulcus plane.

Our study has several limitations. First of all, this study was conducted with retrospective data including patients with not clearly defined inclusion criteria. As such, there were eyes with high astigmatism. as well as eyes post-refractive surgery, in the data. Secondly, the small number of eyes and small variability of sulcus sizes limit the significance of the results. Lastly, the largest limitation is the lack of a control group using ultrasound imaging or a calibration implant behind the iris. The validity of the method could be checked by measuring the dimensions of an intraocular lens postoperatively, which will be the scope of our next paper. In addition, the validity of OCT scans has already been shown by various other authors [25,26]. We, however, believe that they are not the correct tool for validation as UBM scans suffer from various limitations, too. First, the image quality is extremely dependent on the experience of the examiner. Second, the scan method does not allow us to control the scan direction—the operator is unable to control whether the device is exactly scanning through the center or only along a secant. In contrast, AS-OCT uses image registration along the corneal vertex and, therefore, the direction of the scan can be controlled. The best way to validate this method in our opinion is to measure the known size of an implant in the ciliary sulcus. We will provide such a study in a subsequent publication. In addition, Figure 1 and Figure 3 depict the difference in contrast and resolution between ultrasound and AS-OCT. We will also point out that the image resolution presented in this paper may be insufficient; but artificially increasing the resolution via bicubic resampling may also deteriorate image quality. Original images are available upon request from the corresponding author. We used an optimized parameter set to overcome out-of-the-box image quality of a clinical AS-OCT device. The measurement method itself was simply based on drawing a caliper between two points, which were identified by the examiners to be the location of the cilary sulcus. This method and the parameters can easily be adopted by other scientists. Nevertheless, we believe that the described method will be helpful to improve sulcus measurements for the sizing of phakic IOLs, as well as for the Lumina. Many authors describe the importance of the correct sizing of phakic lenses [4,6,7,8,9,18,36,37]. We analyzed the amount of adverse events for sulcus-fixated phakic IOLs that were associated with sizing errors in the Manufacturer and User Device Experience Facility (MAUDE) database (https://www.accessdata.fda.gov/scripts/cdrh/cfdocs/cfmaude/search.cfm, accessed on 23 January 2023) and found that sizing error files approximately 40% of all events (data on file). However, it is unknown whether these cased were based on VHF or OCT sizing. Therefore, there is a great demand in refractive surgery for an improvement in ICL sizing [1]. Yokoyama reported a 95% confidence interval for the limit of agreement for STS measurements with VHF imaging between −0.79 mm and 1.25 mm. They pointed out that inter-examiner/inter-rater variability should be considered for ICL sizing [19]. With our method, the variability (95% confidence interval) ranged from −0.08 mm to 0.18 mm, which is significantly more precise than the values reported for ultrasound. One reason for the high variability of ultrasound measurements was the lack of a controlled measurement position. On the other hand, the OCT is equipped with an eye tracker that centers the scans over the pupil’s center, allowing the examiner to choose the direction and position of the scan (if the meridional goes through the center or is off-center). UBM software shows an indicator of this position but without an angular value (compare Figure 1, which is less accurate that the predefined angular scan in the OCT. The Lumina IOL provides approximately four-dimensional refractive change with a lateral compression of 0.6 mm. Therefore, the precision required for the Lumina is approximately 0.1 mm, as this corresponds to an accommodative change of approximately 0.67 D—corresponding to a miscalculation of 0.1 mm (too small), which may result in a loss of 0.67 D (16.7%) in accommodation. Given the reproducibility of ultrasound imaging, a 100% loss of accommodation (if the lens is <0.6 mm too small) or a compression of ciliary tissue (if the lens is too large) has to be considered, which is unacceptable in both cases. Our future investigations of postoperative images will reveal if the preoperative sizing method and the manufacturing precision yield the intended lens fit.

## 4. Conclusions

In conclusion, AS-OCT is a useful, easy, and reliable method for measuring the dimensions of the ciliary sulcus by measuring the distance from pigment to pigment. However, the validity and accuracy of this method still require investigation. Future clinical studies using this method should provide additional data on the validity and clinical potential. Ultrasound may still provide a deeper insight into anatomical structures; however, the method is burdensome to the patient, time-consuming, requires experienced staff, and needs equipment such as immersion cups and immersion gel. In addition, the ultrasound probes are extremely sensitive and degrade over time. This makes ultrasound a rather expensive method in the long term. Non-contact measurements using AS-OCT are less burdensome for patients, can be performed by less experienced staff, are less time-consuming, do not need consumables, and allow for circumferential assessments of the ciliary sulcus. Thus, we believe that AS-OCT has great potential to supersede ultrasound in the assessment of the ciliary sulcus in standard situations, such as during sulcus implantation.

## Figures and Tables

**Table 1:** Scan parameters used for sulcus measurement. Scan direction was varied between 0°, 30° and 150°.

Setting	Value
Scan Method	2D Single
Focus	Anterior Segment
B-Scan Range	16 mm
A/B Scan	2000
Slice Repeat	64
Scan Duration	2.69 s

**Table 2:** Preoperative patient characteristics (IOLMaster).

Parameter	Unit	Mean ± SD [Range]	*p* Value *
Mean K (IOLMaster)	D	43.60 ± 2.15 [38.375–47.475]	*p* < 0.001
Mean K (CASIA2)		43.72 ± 2.12 [38.79–47.60]	
Keratometric astigmatism (IOLMaster)		0.75 ± 0.65 [0–4.25]	*p* < 0.553
Keratometric astigmatism (CASIA2)		0.70 ± 0.49 [0.04–2.90]	
ACD (IOLMaster)	mm	3.24 ± 0.38 [2.26–4.07]	*p* < 0.001
ACD (CASIA2)		3.36 ± 0.35 [3.29–4.20]	
AL		23.72 ± 1.03 [20.65–26.06]	-/-
WTW		11.98 ± 0.47 [11.0–13.4]	-/-

* paired Wilcoxon test.

**Table 3:** Mean and SD of the sulcus measurement and automated anterior chamber parameters. All values are in millimeters.

Value	Reader 1	Reader 2	R^2^/*p*-Value
STS	11.63 ± 0.54 [10.66–12.83]	11.64 ± 0.52 [10.70–12.89]	R^2^ = 0.986, *p* = 0.0167
SPD	4.17 ± 0.28 [3.75–4.97]	4.16 ± 0.28 [3.73–5.0]	R^2^ = 0.965, *p* = 0.337
ACW	11.72 ± 0.44 [10.76–12.74]	-/-	
ATA	11.56 ± 0.40 [10.69–12.47]	-/-	

## Data Availability

Data are contained within the article.
